# Knowledge of Targeted Muscles and Proper Form in Strength Training: A Cross-Sectional Survey of 1000 Adults Across Age, Sex, and Instructional Experience

**DOI:** 10.3390/sports13090322

**Published:** 2025-09-11

**Authors:** Yoshiki Kobayashi, Megumi Gonno, Kyosuke Oku, Yuki Mori, Noriyuki Kida

**Affiliations:** 1 Enjoydream Holdings, Inc., 201-5-23 Koshien Urakaze-cho, Nishinomiya-shi 663-8165, Hyogo, Japan; kobayashi-trainers@ab.auone-net.jp; 2 Department of Childhood Education, Faculty of Childhood Education, Nagoya Aoi University, 3-40 Shioji-cho, Mizuho-ku, Nagoya-shi 467-8610, Aichi, Japan; gonno@nagoya-aoi.ac.jp; 3 Faculty of Arts and Sciences, Kyoto Institute of Technology, Hashikami-cho, Matsugasaki, Sakyo-ku, Kyoto-shi 606-8585, Kyoto, Japan; kyosuke.oku@gmail.com; 4 Department of Child Studies, Faculty of Child Studies, Sonoda University, 7-29-1 Minami Tsukaguchi-cho, Amagasaki 661-8520, Hyogo, Japan; y_mori@sonoda-u.ac.jp

**Keywords:** training literacy, exercise form and movement, targeted muscle knowledge

## Abstract

With the growing availability of strength training information through online platforms and social media, there is an increasing need to ensure that individuals possess sufficient knowledge to train safely and effectively. Nonetheless, previous studies on strength training literacy have primarily focused on university students and have not adequately examined differences in knowledge across age groups or the roles of training experience and instruction. This study aimed to assess the knowledge of (a) targeted muscle groups and (b) proper form and movement among adults aged 20–69 years and to explore how this knowledge varies by age, sex, and experience in training and professional instruction. A total of 1000 adults (100 males and 100 females in each decade from their 20s to 60s) participated in an online survey. The participants were categorized into three groups according to their training and instructional experience. Knowledge was assessed using 10 items pertaining to targeted muscles and 18 items related to exercise form and movement. Three-way analysis of variance was conducted to analyze the associations between age, sex, and experience. Overall, 75.5% of the participants reported strength training experience, whereas 29.3% had received instruction. Knowledge of the targeted muscle groups was significantly higher in participants in their 60s than in those in their 20s (*p* = 0.014); however, the overall accuracy remained below 60%. No significant effect of instruction on anatomical knowledge was observed. In contrast, both training and instructional experience were positively associated with knowledge of form and movement, although the accuracy remained modest among all groups (50–60%). Sex differences in instructional experience varied by age, with older females reporting higher rates than their male counterparts. In conclusion, knowledge of strength training among adults remains insufficient, particularly regarding anatomical targets. Although instruction enhances the understanding of form and movement, it does not guarantee anatomical literacy. To improve training outcomes and safety, instructional strategies should integrate clear, structured, and pedagogically informed approaches that emphasize movement execution and muscle engagement.

## 1. Introduction

Over the past decades, the means of acquiring strength training knowledge have shifted from printed resources such as books and magazines, and later from television programs and DVDs to digital platforms. This transition has made training information more accessible than ever. In recent years, the availability of such information has further expanded beyond these traditional media to include video platforms, social media, and various websites (e.g., YouTube, Facebook). Importantly, these digital platforms have also enabled individuals—not only experts or publishers—to share training knowledge, marking a notable step in their evolution. While this has promoted broader engagement in strength training, it has also raised concerns regarding variability in information quality and reliability [1,2]. Individuals lacking adequate knowledge may accept inaccurate information uncritically and perform exercises based on it. Consequently, they may fail to gain their intended benefits and face an increased risk of injury due to improper form or technique.

To perform strength training safely and effectively, simply receiving information is insufficient. Individuals must also possess the ability to interpret and apply it accurately. In this regard, understanding fundamental principles of resistance training is indispensable. These include the principle of progressive overload and the manipulation of key training variables—volume, intensity, and frequency—to achieve specific goals related to strength, hypertrophy, or muscular endurance [3,4,5,6,7]. Additional principles such as specificity, consistency, and periodization are essential for long-term progress and injury risk reduction [3,4,5,6,7]. Moreover, muscle growth and strength gains depend not only on resistance exercise but also on adequate sleep, nutrition, and rest [3,4,5,6,7].

Differentiating compound lifts (e.g., squat, bench press, deadlift) from single-joint exercises is also important for safe and effective training initiation. Before applying these broader principles, individuals should understand which muscles each exercise targets and how to perform movements with proper form. Hence, assessing the current state of such knowledge and exploring its association with factors such as training experience and instruction are both essential. These insights are crucial in shaping future educational strategies and training practices.

Previous studies [8,9,10,11,12,13] on knowledge related to strength training have primarily focused on university students. For instance, Oshita et al. [9,10] asked participants to identify the primary muscles used in specific exercises such as squats and found that many identified the quadriceps muscles. Notably, even among those with regular exercise habits, approximately half failed to recognize the gluteal muscles as a primary target, and some selected unrelated areas such as the neck or shoulders. Similarly, Kobayashi et al. [8] reported that nearly 40% of students with strength training experience mistakenly identified lat pulldown, a back exercise, as targeting the chest. These findings consistently indicate limited strength training literacy among young adults, particularly university students, even among those with prior training experience, and suggest that professional instruction may play a critical role in fostering accurate anatomical understanding.

Although these studies were limited to both age range and focus, it remains unclear whether similar patterns hold across other age groups. They primarily examined anatomical knowledge, whereas critical components such as proper form and movement, which are closely linked to safety and effectiveness, were largely overlooked. For instance, older individuals may benefit from greater life experiences or lack sufficient exposure to strength training. Examining how knowledge and instruction interact across a broader age range is crucial for developing effective educational strategies that respond to diverse populations. 

Against this background, the present study targeted a broad age range of adults, from their 20s to 60s, with the following two main objectives: (1) to examine knowledge of the targeted muscle groups in strength training and (2) to assess the knowledge pertaining to proper form and movement, which are critical for the safe and effective execution of strength exercises. To facilitate these assessments, representative muscle areas such as the chest, quadriceps, back, and hamstrings were selected, and the participants’ knowledge was measured using typical exercises associated with each area. The knowledge of form and movement was evaluated using representative exercises based on visual materials. Additionally, to allow for a more precise analysis of the association between knowledge and experience, participants were categorized into three groups according to their training history and experience of receiving instruction.

We hypothesized that individuals with training experience—particularly those who had received instruction—would demonstrate higher knowledge scores regarding targeted muscle groups, as well as proper form and movement, compared with individuals without such experience. We further expected that age and sex would be associated with differences in knowledge levels.

## 2. Materials and Methods

### 2.1. Study Design and Participants

This study employed a cross-sectional design and conducted an online survey platform. Participants were recruited through a commercial survey company (Cross Marketing Inc., Tokyo, Japan) that distributed survey invitations to individuals registered on the online panel. The recruitment targeted adults in their 20s to 60s, stratified by age and sex. Specifically, 100 males and 100 females were recruited from each decade-based age group (20s, 30s, 40s, 50s, and 60s), resulting in a total sample size of 1000 participants (*n* = 1000).

Recruitment was closed once these quotas were filled. While this quota sampling ensured balanced distributions across age and sex, the use of a commercial panel does not guarantee full representativeness of the general population. Inclusion criteria were age 20–69 years, residence in Japan. Exclusion criteria included incomplete responses or clearly inconsistent response patterns (e.g., choosing the same option for all items without variation). Prior to answering the questionnaire, the participants were presented with an informed consent form on the initial page of the web-based survey. Only those who provided informed consent were permitted to participate. The study protocol was conducted in accordance with the Declaration of Helsinki and approved by the Research Ethics Committee of Kyoto Institute of Technology (approval no: 2023-55, date: 2 October 2023).

### 2.2. Measures

The survey consisted of questions designed to assess knowledge of (a) targeted muscle groups and (b) proper form and movement during strength training. In addition, participants were asked about their training experience and their experience receiving instructions.

#### 2.2.1. Assessment of Knowledge of Targeted Muscle Groups

Knowledge of the targeted muscle groups was assessed using 10 items derived from four representative exercises: squat, leg extension, lat pulldown, and push-up. For each item, participants responded using a five-point Likert scale: “strongly agree,” “somewhat agree,” “neither agree nor disagree,” “somewhat disagree,” and “strongly disagree.” Responses of “strongly agree” and “somewhat agree” were categorized as affirmative, while “somewhat disagree” and “strongly disagree” were categorized as negative. Responses of “neither agree nor disagree” were excluded from the calculation, as they did not indicate correct knowledge. When participants selected either an affirmative or negative response, their answers were evaluated as correct or incorrect, respectively, based on the corresponding target muscle group. The total score ranges from 0 to 10, with one point awarded for each correct response.

#### 2.2.2. Assessment of Knowledge of Proper Form and Movement

Knowledge of proper form and movement was assessed using 19 items based on the same four exercises used to assess knowledge of the targeted muscle groups: squat, leg extension, lat pulldown, and push-up. One item was excluded from the analysis because it was not directly related to form or movement, resulting in 18 items included in the final analysis. The evaluation method was identical to that used for the knowledge of the targeted muscle groups, as described in section (a). Items were developed with reference to the official textbook of the National Strength and Conditioning Association (NSCA) [14] and were customized to fit the aims of this study. The item pool was initially developed and reviewed by the first author, who has practical experience in strength training instruction, and was further refined in discussion with co-authors to ensure clarity and appropriateness. This process ensured face validity.

#### 2.2.3. Training Experience and Experience of Receiving Instruction

**Figure 1:**
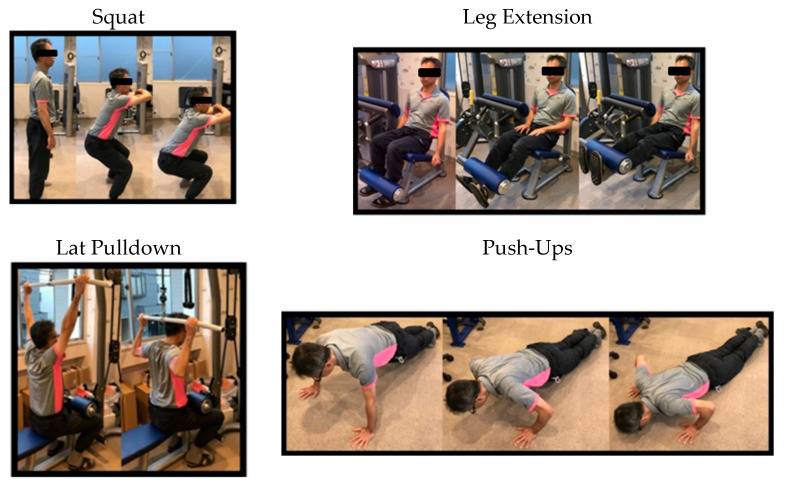
Demonstration of exercises included in the questionnaire.

To assess the participants’ strength training experience, the survey included eight items related to past engagement in training and instructional contexts (e.g., the use of free weights, resistance machines, participation in group classes or personal training, attendance at lectures, and receipt of professional instruction). In addition, participants were shown photographs of four representative exercises—squat, leg extension, lat pulldown, and push-up (Figure 1—and asked to select one of five response options: “currently doing,” “have done in the past,” “have not done but know the exercise,” “have seen it but don’t know the details,” and “completely unfamiliar.” Participants who answered “yes” to any of the eight training/instruction items or selected either “currently doing” or “have done in the past” for any of the four exercises were classified as having strength training experience. 

Five items were included to assess the participants’ experience of receiving instructions. The participants were asked whether they had ever received instruction in strength training, received instruction from a professional (e.g., a trainer or fitness instructor), attended a lecture or seminar, received personal training, or participated in studio-based group classes. Each item used a binary response format (“yes” or “no”). Participants who answered “yes” to any of these items were classified as having received instructions. This classification was based on the participants’ subjective judgments, regardless of the frequency, content, or format of instruction.

Based on the combination of training experience and experience receiving instruction, participants were categorized into the following three groups: no training experience, training experience without experience receiving instruction, and training experience with experience receiving instruction. 

The full list of assessment items is provided in Table S1.

### 2.3. Statistical Analysis

First, cross-tabulations by age group and sex were conducted to examine differences in the distribution of strength training experience and experience of receiving instruction. The chi-square (χ^2^) test of independence was then performed. When significant differences were found, adjusted residuals were examined to identify specific group differences. In addition, effect sizes were calculated using the phi coefficient.

Subsequently, two separate three-way analyses of variance (ANOVAs) were conducted to analyze the participants’ knowledge of (a) targeted muscle groups and (b) proper form and movement. In both analyses, the dependent variable was the number of correct responses. The between-subject factors were age group (five levels: 20s, 30s, 40s, 50s, and 60s), sex (male, female), and training experience (three groups: no training experience, training experience without experience of receiving instruction, and training experience with experience of receiving instruction).

When significant interactions were detected, simple main effects were examined to explore specific group differences. For significant main effects, Bonferroni correction was applied to adjust for multiple comparisons. Effect sizes for the ANOVAs were calculated using eta squared (η^2^) to assess the magnitude of observed differences.

All statistical analyses were performed using IBM SPSS Statistics version 27 (IBM Corp., Armonk, NY, USA). The significance level was set at *p* < 0.05.

## 3. Results

### 3.1. Participant Characteristics and Distributions of Training Experience

**Figure 2:**
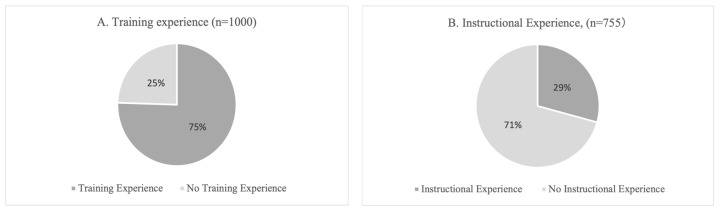
Distribution of training and instructional experience among participants. (**A**) Training experience among all participants (*n* = 1000; Male *n* = 500, Female *n* = 500). (**B**) Instructional experience among participants with training experience (*n* = 755). Values represent percentages of participants in each category. Training Experience = participants who reported engaging in strength training; Instructional Experience = participants with training experience who had received professional instruction.

**Figure 3:**
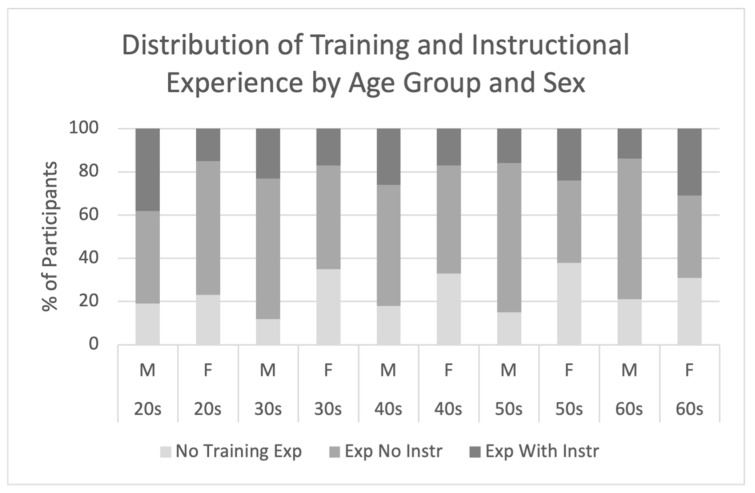
Distribution of training and instructional experience by age group and sex. (*n* = 1000; Male *n* = 500, Female *n* = 500). Bars represent the proportions of participants in each category. No Training Exp = participants without strength training experience; Exp No Instr = participants with training experience but without professional instruction; Exp With Instr = participants with training experience and professional instruction.

Out of 1000 participants, 755 (75.5%) reported having strength training experience (415 males and 340 females). Among those with training experience, 221 (29.3%) reported having experience of receiving instructions. The overall distributions of training and instructional experience are illustrated in Figure 2, and their breakdowns by age group and sex are presented in Figure 3.

Based on the combination of training experience and experience receiving instruction, participants were categorized into three groups: (1) no training experience, (2) training experience without experience receiving instruction, and (3) training experience with experience receiving instruction. The proportions of these groups according to age and sex are shown in Table 1. When the two groups were combined according to training experience (with and without experience in receiving instruction), the overall proportion of participants with training experience was approximately 80% among males and 70% among females across all age groups.

To examine the effects of age and sex on the distribution of training experience and experience of receiving instructions, cross-tabulations were conducted, followed by the chi-square test of independence. The results revealed significant differences by age group for both males and females (χ^2^ = 25.77, *p* = 0.001). The adjusted residuals indicated that, among males, the proportion of those with training experience and experience of receiving instruction was significantly higher in their 20s and significantly lower in their 50s and 60s. Among females, the proportion of those with training experience but without experience of receiving instruction was significantly higher in their 20s, whereas the proportion of those with no training experience was significantly lower. In contrast, in the 60s group, the proportion of females with training experience and experience of receiving instruction was significantly higher than in the other age groups.

A cross-tabulation analysis also revealed a significant sex difference (χ^2^ = 20.97, *p* = 0.007). Adjusted residuals showed that in the 20s group, females were significantly more likely to report training experience without receiving instructions, while males were significantly more likely to report both training experience and receiving instructions. In the 30s–50s group, females were significantly more likely to have no training experience, whereas males were more likely to have training experience without receiving instruction. In the 60s group, females were significantly more likely to report both training experience and experience of receiving instruction.

Further analysis of sex differences in the experience of receiving instruction by age group showed a notable trend: in the 20s group, 46.9% of males had received instruction compared to only 9.5% of females. Conversely, in the 50s and 60s groups, the proportion of males who had received instruction was the lowest at 18.8% and 17.7%, respectively, whereas the corresponding proportions among females were higher at 38.7% and 44.9%, respectively. This finding suggests a reversal of the sex gap in instructional experience among the older age groups. 

### 3.2. Results: Knowledge of Targeted Muscle Groups

Table 2 presents the means and standard deviations for the number of correct responses to the 10 questions assessing participants’ knowledge of the muscle groups targeted by specific strength training exercises, categorized by age group, sex, and experience of training and receiving instruction.

Three-way ANOVA was conducted with age group (five levels: 20s–60s), sex (male, female), and training experience (three groups: no training experience, training experience without experience of receiving instruction, and training experience with experience of receiving instruction) as the between-subject factors.

No significant three-way or two-way interactions involving age were found (age × sex × experience: F(8, 970) = 1.117, *p* = 0.349, η^2^ = 0.009; age × sex: F(4, 970) = 0.251, *p* = 0.909, η^2^ = 0.001; age × training experience: F(8, 970) = 1.775, *p* = 0.078, η^2^ = 0.014). However, there was a significant main effect of age group (F(4, 970) = 2.612, *p* = 0.034, η^2^ = 0.011). Post hoc comparisons using the Bonferroni method revealed that participants in their 60s (M = 5.80, SD = 2.04) scored significantly higher than those in their 20s (M = 5.42, SD = 2.20, *p* = 0.014).

A significant interaction between sex and training experience was also observed (F(2, 970) = 13.492, *p* < 0.001, η^2^ = 0.027). A simple main effects analysis indicated that among participants with no training experience, females (M = 5.08, SD = 2.43) scored significantly higher than males (M = 3.80, SD = 2.56, *p* < 0.001). In contrast, no significant sex differences were found among participants with training experience, regardless of whether they had received instruction (without instruction: *p* = 0.070; with instruction: *p* = 0.178).

Furthermore, within each sex, participants with training experience scored significantly higher than those with no training experience (males, *p* < 0.001; females, *p* = 0.001). However, among participants with training experience, the presence or absence of experience in receiving instructions was not associated with significant differences in scores (males, *p* = 0.154; females, *p* = 0.333). These results are summarized in Table 3.

### 3.3. Knowledge of Proper Form and Movement

Table 4 shows the means and standard deviations for the number of correct responses to 18 questions assessing knowledge related to the appropriateness of form and movement during strength training exercises, categorized by age group, sex, and experience of training and receiving instructions.

Three-way ANOVA was conducted with age group (five levels), sex (male, female), and training experience (three groups: no training experience, training experience without experience of receiving instruction, and training experience with experience of receiving instruction) as the between-subject factors.

No significant interactions involving age were found (age × sex × training experience: F(8, 970) = 0.599, *p* = 0.779, η^2^ = 0.005; age × sex: F(4, 970) = 0.254, *p* = 0.907, η^2^ = 0.001; age × training experience: F(8, 970) = 1.261, *p* = 0.260, η^2^ = 0.010). In addition, the main effect of age group was not significant (F(4, 970) = 0.358, *p* = 0.838, η^2^ = 0.001), indicating no substantial age-related differences in form and movement knowledge.

However, a significant interaction was found between sex and training experience (F(2, 970) = 4.658, *p* = 0.010, η^2^ = 0.010), prompting a simple main effects analysis. Among the participants with no training experience, females scored significantly higher than males (*p* = 0.049). In contrast, among those with both training experience and experience in receiving instructions, males outperformed females (*p* = 0.033). No significant sex difference was found among participants with training experience but no experience of receiving instruction (*p* = 0.137).

Further comparisons across training experience groups within each sex revealed that for both males and females, participants with training experience but no experience of receiving instruction scored significantly higher than those with no training experience (males, *p* < 0.001; females, *p* < 0.001). Moreover, those with both training experience and experience of receiving instruction scored significantly higher than those with training experience but no instruction (males, *p* < 0.001; females, *p* = 0.015). A summary of the three-way ANOVA results for knowledge of proper form and movement is presented in Table 5.

## 4. Discussion

### 4.1. Strength Training and Instructional Experience by Age and Sex

This study revealed that 75.5% of the respondents reported engaging in strength training at some point in their lives. Across all age groups, approximately 80% of males and 70% of females had such experience, suggesting that strength training is relatively widespread regardless of sex or age. However, this classification is based on lifetime experience rather than frequency or continuity, which may partially explain the high percentages. By contrast, a national survey conducted by the Sasakawa Sports Foundation [15] reported that only 12% of Japanese individuals engage in strength training at least once a year, indicating that strength training has not yet been established as a regular part of their daily lives. 

Among those with training experience, approximately 30% reported having received instruction, a proportion comparable to that observed in previous research among university students [14]. Notably, this study provides valuable insights into the instructional experiences of adults in their 30s and older, a population that has been less frequently examined in prior work. The findings showed that among individuals in their 50s and 60s, the proportion of those with instructional experience was lower for males but higher for females than for other age groups.

Among participants in their 50s and 60s, a greater proportion of women reported having received instruction compared to men. Notably, this sex difference does not appear to be attributable to disparities in overall strength training experience, as both sexes exhibited comparable levels of training engagement. Data from the present study revealed that men in their twenties had significantly more instructional experience than women in the same age group. This earlier exposure to guidance may have diminished their perceived need for subsequent instruction in later adulthood, thereby contributing to the observed disparity in instructional experience. However, it should be noted that the current data reflect the experiences of today’s younger generation, and it remains unclear whether men who are now in their 50s and 60s had similarly greater access to instruction in their twenties. Therefore, caution is warranted when drawing generational comparisons or inferring causal relationships based on these findings.

### 4.2. Discussion: Knowlwdge of Targeted Muscle Groups

This study found that participants in their 60s scored significantly higher than those in their 20s on knowledge of the muscle groups targeted by specific strength training exercises. However, no significant differences were observed among those in their 20s and 50s. Previous studies focusing on university students have reported insufficient knowledge and misconceptions regarding the relationship between training exercises and targeted muscle groups [8,9,10,11,12,13]. The present study extends these findings by encompassing a broader age range from 20s to 60s, thereby offering a more comprehensive view of age-related trends in training-related knowledge.

The relatively higher scores observed among individuals in their 60s may reflect increased health awareness and interest in physical activity for disease prevention, which is commonly observed with aging. In addition, recent improvements in access to training information through online video platforms and social media may have facilitated the acquisition of basic training knowledge among older adults. Despite this, the overall accuracy remained modest; scores among participants in their 20s through 50s hovered at around 50%, and even among those in their 60s, the rate of correct answers did not exceed 60%. These results indicate that knowledge of the targeted muscle groups is generally limited across all age groups.

Inaccurate knowledge of the targeted muscle groups can result in the selection of inappropriate exercises, potentially leading to mismatches between training objectives and execution. This, in turn, may compromise the effectiveness of training and increase the risk of injury owing to the poor form or overload of the unintended muscle groups.

Participants with training experience demonstrated significantly higher accuracy in identifying targeted muscle groups, a finding consistent with those of previous studies [8,9]. This suggests that engaging in training may enhance an individual’s awareness of the relationship between exercise and muscle groups. However, even among participants with training experience, the correct answer rates remained at approximately 60%, highlighting that experiential learning alone may not be sufficient for acquiring accurate and consistent knowledge.

By contrast, no significant difference in knowledge accuracy was found based on whether the participants had received professional instruction. This suggests that receiving instruction alone does not necessarily translate into higher anatomical literacy. Supporting this interpretation, prior research has shown that even individuals who received instructions continued to exhibit low accuracy when asked to identify the muscles targeted by basic exercises such as push-ups and squats [8]. The present study reinforces this notion, revealing no appreciable difference in knowledge across instructional experience groups regardless of age. These findings imply that, while instruction may play a role in guiding behavior or motivation, its impact on foundational knowledge acquisition may be limited unless coupled with targeted educational interventions.

### 4.3. Knowledge of Training Form and Movement

This study found no significant age-related differences in knowledge of proper training forms and movements. While previous studies have examined anatomical knowledge among university students [8,10], few have addressed form- and movement-related literacy across a wide range of age groups. The present study is, therefore, novel in that it provides an overview of the current knowledge levels of individuals in their 20s through 60s, offering valuable insights into the development of future training education and instructional strategies. However, overall accuracy remained below 50% across all age groups, with a mean score of approximately 47%. These findings suggest that literacy regarding training forms and movements is inadequate. A lack of such knowledge may compromise training effectiveness and increase the risk of injury due to improper technique.

By contrast, participants with strength training experience scored significantly higher than those without. Forms and movements are typically acquired through repeated practice accompanied by proprioceptive and visual feedback. This suggests that practical experience may contribute to improved knowledge. Nevertheless, even among those with training experience, the accuracy remained at around 50%, indicating that experience alone was insufficient for developing adequate literacy. Therefore, training experience should be complemented by more structured and continuous knowledge support to enhance the understanding of correct forms and movements.

Furthermore, participants with experience receiving instructions demonstrated significantly higher levels of knowledge than those without such experience. This implies that instruction plays an important role in promoting accurate understanding. Since form and movement are visually observable during training, instructors can provide real-time feedback, enabling more effective error correction. Oshita et al. [10] emphasized the importance of receiving professional guidance to prevent injuries and ensure correct execution. In contrast, the present study found no significant difference in anatomical knowledge (i.e., knowledge of the targeted muscle groups) between those with and without instructional experience. This may be due to the inherently less observable nature of anatomical targeting; trainers may not detect such misconceptions unless they are explicitly reported by trainees. Consequently, instructions may prioritize an observable form over internal muscle activation. 

It is also notable that even among those who received instruction, the average accuracy of form and movement remained at approximately 60%. This indicates that, while instruction supports knowledge acquisition, it does not guarantee sufficient understanding. To improve training literacy effectively, instructional practices should include well-structured explanations, step-by-step demonstrations, and concrete examples that facilitate comprehension. A shift toward more intentional and pedagogically informed approaches may be necessary to bridge this knowledge gap. 

### 4.4. Implications for Strength Training Instructors

This study found that, while knowledge of training form and movement was positively associated with both training and instructional experience, knowledge regarding the specific muscle groups targeted by exercises remained insufficiently developed across all participant groups. One possible explanation is that in practical training settings, instructions tend to prioritize correcting observable movement patterns rather than fostering an anatomical understanding of muscle activation. 

In typical instructional scenarios, trainers often begin by explaining which muscles should be engaged in a given exercise, followed by demonstrations of the correct form. However, even when trainees successfully replicate the instructed movements, they may still experience uncertainty regarding which muscles are actually being used. In such cases, trainers may employ supplementary techniques such as visual modeling or tactile cues to highlight the intended muscle activation. Nevertheless, many trainees reported difficulty developing sensory awareness of the targeted muscle areas. 

These challenges can result in instructions that become overly routine and mechanistic, with a limited impact on conceptual understanding or perceived training efficacy. To address this issue, it is essential that trainers move beyond purely prescriptive guidance and actively communicate the rationale behind each exercise, that is, what its intended effects are and which specific muscles it targets. Incorporating explanations that integrate verbal descriptions, visual demonstrations, and sensory feedback can promote a more embodied and meaningful understanding among trainees. 

Furthermore, although access to training-related content has significantly increased through online platforms such as YouTube and social media, this information is typically delivered in a unidirectional manner and lacks opportunities for interactive clarification or corrective feedback. Consequently, misconceptions may persist or be reinforced. Direct in-person instruction remains essential to facilitate accurate comprehension and safe practice, particularly for novice trainees or those lacking prior experience. 

To foster more robust training literacy, instructional strategies should aim not only to improve mechanical execution but also to develop conceptual understanding. Trainers should adopt a pedagogical orientation characterized by deliberate communication, individualized feedback, and structured progression. Such an approach can enhance both the effectiveness and safety of strength training, particularly in the general population with limited prior exposure. 

### 4.5. Limitations

This study has several limitations that should be acknowledged.

First, although it covered a broad age range from the 20s to the 60s, it did not include older adults or teenagers. Thus, the findings cannot be generalized to populations outside this age range.

Second, the sample consisted exclusively of Japanese adults, which may restrict the generalizability of the results to other cultural contexts. Future research should examine diverse populations to provide a more comprehensive understanding.

### 4.6. Future Directions

When interpreting the findings of this study, it is important to consider the individual and contextual factors that may underlie variations in training knowledge and experience. While this study examined whether participants had strength training experience or had received instructions, it did not investigate the broader determinants of these experiences, such as personal interests, lifestyle habits, physical and psychological characteristics, social support systems, or varying levels of awareness across sexes and age groups. For example, the relatively high rate of instructional experience observed among older female participants may be influenced by greater health consciousness or the availability of supportive environments such as community fitness programs. 

In addition, differences in knowledge and behavioral patterns may be shaped by the specific goals associated with training, whether oriented toward health maintenance, athletic performance, or esthetic objectives, such as body shaping. The current study assessed knowledge primarily through a correct/incorrect judgment format but did not address how such knowledge is applied to actual training behavior. Although knowledge is generally regarded as contributing to safe and effective practices, it does not necessarily translate directly into higher-quality execution or outcomes. Individuals may develop appropriate techniques through imitation, embodied feedback, or routine practices, even in the absence of explicit knowledge. Thus, it is essential to critically examine the often-assumed causal link between knowledge acquisition and behavioral quality. 

Future research should adopt a more integrated approach to explore how cognitive, psychological, and social factors affect training-related knowledge. It is also important to assess how such knowledge influences not only behavioral choices but also training outcomes over time. Educational interventions and strategies for behavioral change should be designed and evaluated in this broader context. Longitudinal studies, in particular, could offer insights into how training literacy develops, is retained, and influences ongoing behavior. Moreover, the present study classified instructional experience as present or absent without evaluating the instructional quality, frequency, or expertise of the instructor. These aspects likely substantially influence the depth and accuracy of knowledge retention. Future studies should, therefore, explore the qualitative features of instruction to identify more effective educational practices and optimize training support strategies. In addition, while this study assessed knowledge of targeted muscles and proper form, it did not directly measure muscular activity during exercise. Incorporating such data could provide further insight into the relationship between knowledge and actual muscle engagement.

## 5. Conclusions

This cross-sectional study aimed to clarify the current state of training-related knowledge among adults aged 20–69 years. A key contribution lies in its inclusion of both anatomical knowledge—specifically, understanding which muscles are targeted by particular exercises—and knowledge of the correct form and movement, the latter of which has received less attention in prior research. The participants were classified based on their strength training and instructional experience, allowing for a multifaceted analysis of how such experience relates to their knowledge levels.

The results revealed that individuals with strength training experience demonstrated significantly higher knowledge in both domains, suggesting that such experience contributes to literacy development. Furthermore, those with instructional experience scored higher in knowledge of form and movement, although no such advantage was found in knowledge of the targeted muscle groups. This may indicate that although instruction can enhance the visible performance-related aspects of training, it may not sufficiently address a deeper anatomical understanding.

The significance of this study lies in its comprehensive depiction of training knowledge across sexes, age groups, and experiential backgrounds. Particularly noteworthy is the finding that knowledge regarding targeted muscle groups may be inadequately addressed in the current instructional practice. Moving forward, greater emphasis should be placed on instructional methods that not only improve the form and technique but also clarify the intended muscular targets of each exercise. Instructional strategies should encourage bidirectional communication and utilize clear, structured explanations supplemented by visual and sensory cues. Additionally, future studies should investigate the influence of training objectives, instructional quality, and frequency, as well as exploring how knowledge impacts behavior, using longitudinal and qualitative methodologies that account for psychological and sociocultural factors. 

## Figures and Tables

**Table 1:** Participant training experience by age group and gender (*n* = 1000; male *n* = 500, female *n* = 500). Values represent the number of participants and percentages within each category.

Male	No Exp.			Exp-No Instr.			Exp-Instr.			Total
Age Group	*n*	Adjusted Residuals		*n*	Adjusted Residuals		*n*	Adjusted Residuals		
20s	19	0.6		43	−3.8		38	3.9		100
30s	12	−1.5		65	1.2		23	−0.1		100
40s	18	0.3		56	−0.8		26	0.7		100
50s	15	−0.6		69	2.1		16	−2.0		100
60s	21	1.2		65	1.2		14	−2.5		100
Total	85			298			117			500
					
Female	No Exp.			Exp-No Instr.			Exp-Instr.			Total
Age Group	*n*	Adjusted residuals		*n*	Adjusted residuals		*n*	Adjusted residuals		
20s	23	−2.2		62	3.3		15	−1.6		100
30s	35	0.7		48	0.2		17	−1.0		100
40s	33	0.2		50	0.6		17	−1.0		100
50s	38	1.4		38	−2.1		24	0.9		100
60s	31	−0.2		38	−2.1		31	2.8		100
Total	160			236			104			500
					

Adjusted residuals > |1.96| indicate significant deviation from expected counts (*p* < 0.05). Groups were divided into: –No Experience (No Exp.) = participants without strength training experience; –Experience without Instruction (Exp-No Instr.) = participants with training experience but without professional instruction; –Experience with Instruction (Exp-Instr.) = participants with training experience and professional instruction.

**Table 2:** Mean ± standard deviation of correct responses to anatomical knowledge questions (Q3) by age group, sex, and training experience (*n* = 1000; male *n* = 500, female *n* = 500).

Age Group	Male			Female			Total
	No Exp.	Exp-No Instr.	Exp-Instr.	No Exp.	Exp-No Instr.	Exp-Instr.	
20s	3.11 ± 2.96	6.00 ± 2.05	5.84 ± 1.70	4.70 ± 2.80	5.76 ± 1.63	5.27 ± 1.79	5.42 ± 2.20
30s	3.75 ± 2.49	6.15 ± 1.69	6.26 ± 1.60	5.49 ± 2.16	5.33 ± 2.34	6.24 ± 1.64	5.72 ± 2.06
40s	4.28 ± 2.16	5.93 ± 2.01	6.27 ± 1.25	4.48 ± 2.62	6.04 ± 1.94	6.18 ± 1.91	5.64 ± 2.14
50s	3.47 ± 2.56	5.86 ± 2.00	6.50 ± 1.37	4.76 ± 2.39	5.82 ± 2.02	6.33 ± 1.61	5.57 ± 2.17
60s	4.29 ± 2.57	5.97 ± 1.74	7.29 ± 1.33	5.90 ± 2.07	5.34 ± 2.02	6.23 ± 1.86	5.80 ± 2.04
Total	3.80 ± 2.56	5.98 ± 1.88	6.28 ± 1.54	5.08 ± 2.43	5.67 ± 1.98	6.11 ± 1.77	5.63 ± 2.12

Values represent mean ± standard deviation for number of correct answers (range: 0–10). No significant main effect of instruction was observed (*p* > 0.05), although participants with training experience scored significantly higher than those without (*p* < 0.001). Groups were divided into –No Experience (No Exp.) = participants without strength training experience; –Experience without Instruction (Exp-No Instr.) = participants with training experience but without professional instruction; –Experience with Instruction (Exp-Instr.) = participants with training experience and professional instruction.

**Table 3:** Summary of three-way ANOVA results for anatomical knowledge scores (Q3) among participants (*n* = 1000 adults, stratified by age and sex).

Source of Variation	df	F	*p*-Value	η^2^
Age	4970	2.612	0.034	0.011
Sex	1970	1.794	0.181	0.002
Training Experience	2970	49.748	<0.001	0.093
Age × Sex	4970	0.251	0.909	0.001
Age × Training Experience	8970	1.775	0.078	0.014
Sex × Training Experience	2970	13.492	<0.001	0.027
Age × Sex × Experience	8970	1.117	0.349	0.009

df = degrees of freedom; η^2^ = partial eta squared.

**Table 4:** Mean ± SD of correct responses to questions on form and movement (Q4 and Q5) by gender, age group, and training experience (*n* = 1000; male *n* = 500, female *n* = 500).

	Male			Female			Total
	No Exp.	Exp-No Instr.	Exp-Instr.	No Exp.	Exp-No Instr.	Exp-Instr.	
20s	4.26 ± 3.77	9.77 ± 4.24	10.95 ± 3.33	6.43 ± 4.71	9.48 ± 3.55	9.67 ± 4.13	8.99 ± 4.30
30s	5.75 ± 4.49	9.94 ± 4.27	11.65 ± 2.90	7.17 ± 3.94	8.60 ± 4.03	9.59 ± 3.78	9.05 ± 4.23
40s	6.39 ± 4.77	9.02 ± 4.82	12.19 ± 3.48	6.48 ± 4.47	9.36 ± 4.20	10.29 ± 3.55	8.97 ± 4.65
50s	5.33 ± 5.67	9.17 ± 4.88	11.63 ± 4.77	5.63 ± 5.10	7.95 ± 3.86	11.75 ± 2.88	8.49 ± 5.02
60s	5.86 ± 4.75	8.65 ± 5.19	11.21 ± 4.53	7.71 ± 5.29	8.29 ± 3.65	9.77 ± 4.96	8.50 ± 4.93
Total	5.51 ± 4.64	9.28 ± 4.72	11.49 ± 3.63	6.66 ± 4.72	8.84 ± 3.87	10.27 ± 4.03	8.80 ± 4.63

Values represent mean ± standard deviation of correct responses (score range: 0–10). No significant main effect of instruction was found (*p* > 0.05), but participants with training experience scored significantly higher than those without (*p* < 0.001). Groups were divided into –No Experience (No Exp.) = participants without strength training experience; –Experience without Instruction (Exp-No Instr.) = participants with training experience but without professional instruction; –Experience with Instruction (Exp-Instr.) = participants with training experience and professional instruction.

**Table 5:** Summary of three-way ANOVA results for Form and Movement scores among participants. (*n* = 1000 adults, stratified by age and sex).

Source of Variation	df	F	*p*-Value	η^2^
Age	4970	0.358	0.838	0.001
Sex	1970	0.583	0.445	0.001
Training Experience	2970	64.607	0.000	0.118
Age × Sex	4970	0.254	0.907	0.001
Age × Training Experience	8970	1.261	0.260	0.010
Sex × Training Experience	2970	4.658	0.010	0.010
Age × Sex × Experience	8970	0.599	0.779	0.005

df = degrees of freedom; η^2^ = partial eta squared.

## Data Availability

The data presented in this study are openly available in [Figshare] [https://doi.org/10.6084/m9.figshare.29484869].

## Supplementary Materials

The following supporting information can be downloaded at: https://www.mdpi.com/article/10.3390/sports13090322/s1, Table S1: Survey Items Assessing Knowledge of Targeted Muscle Groups and Exercise Form/Movement.
