# Supplementary material for: Knowledge of Targeted Muscles and Proper Form in Strength Training: A Cross-Sectional Survey of 1000 Adults Across Age, Sex, and Instructional Experience
Source: Sports (Basel). 2025 Sep 11;13(9):322. doi: 10.3390/sports13090322 (PMC12473838; doi:10.3390/sports13090322)
Supplement: Supplementary file 1 [file sports-13-00322-s001.zip › sports-3783511-supplementary.pdf]

Table S1. Survey Items Assessing Knowledge of Targeted Muscle Groups and Exercise Form/Movement

---

Q3: Knowledge of Targeted Muscle Groups

Participants evaluated the following statements regarding which muscle groups are primarily activated by each strength-training exercise:

- Squats primarily target the quadriceps (front thigh muscles).
- Squats primarily target the gluteal muscles.
- Squats primarily target the hamstrings (rear thigh muscles).
- Leg extensions primarily target the quadriceps.
- Leg extensions primarily target the gluteal muscles.
- Lat pulldowns primarily target the latissimus dorsi (back muscles).
- Lat pulldowns primarily target the pectoralis major (chest muscles).
- Lat pulldowns primarily target the biceps.
- Push-ups primarily target the pectoralis major.
- Push-ups primarily target the biceps.

Q4/Q5: Knowledge of Proper Exercise Form and Movement

Participants evaluated the correctness of the following statements regarding proper technique and form:

(Squats)

- The knees and toes should point in the same direction.
- The knees should not extend past the toes during the downward motion.
- During descent, the gaze should be directed forward or slightly upward.
- The descent should be performed more slowly than the ascent.
- The ascent should utilize body momentum.
- To improve maximal strength, the load should induce failure within approximately 15 repetitions.(This question was rejected.)

(Leg Extensions)

- The movement should begin with the knees flexed beyond 90 degrees.
- When the exercise becomes challenging, lifting the hips or lower back to complete repetitions is acceptable.
- The flexion phase (returning the weight) should be slower than the extension phase.
- Inhale during knee extension and exhale during flexion.
- Pause briefly at full knee extension.

(Lat Pulldowns)

- The bar should be pulled down to the base of the neck.
- Multiple joints are involved in the movement.
- The return phase should be performed more slowly than the pulling phase.
- The torso should assist the downward movement by generating momentum.

(Push-Ups)

- Hands should be positioned at or slightly below clavicle level.
- The body should remain aligned in a straight line throughout the movement.
- Altering hand width changes the primary muscle groups being targeted.
- The upward (pushing) phase should be slower than the downward phase.
